# Pharmacotherapy from Pre-COVID to Post-COVID: Longitudinal Trends and Predictive Indicators for Long COVID Symptoms

**DOI:** 10.3390/biomedicines12122694

**Published:** 2024-11-26

**Authors:** Nadia Baalbaki, Sien T. Verbeek, Harm Jan Bogaard, Jelle M. Blankestijn, Vera C. van den Brink, Merel E. B. Cornelissen, Jos W. R. Twisk, Korneliusz Golebski, Anke H. Maitland-van der Zee

**Affiliations:** 1 Department of Pulmonary Medicine, Amsterdam UMC, 1105 AZ Amsterdam, The Netherlands; 2 Amsterdam Institute for Infection and Immunity, 1105 AZ Amsterdam, The Netherlands; 3 Amsterdam Public Health, 1105 AZ Amsterdam, The Netherlands; 4 Amsterdam Cardiovascular Sciences Research Institute, Amsterdam UMC, 1105 AZ Amsterdam, The Netherlands; 5 Department of Epidemiology and Data Science, Amsterdam UMC, 1105 AZ Amsterdam, The Netherlands

**Keywords:** precision medicine, long COVID, COVID-19, pharmacotherapy, longitudinal trends

## Abstract

Background/objectives: A significant number of COVID-19 cases experience persistent symptoms after the acute infection phase, a condition known as long COVID or post-acute sequelae of COVID-19. Approved prevention and treatment options for long COVID are currently lacking. Given the heterogeneous nature of long COVID, a personalized medicine approach is essential for effective disease management. This study aimed to describe trends in pharmacotherapy from pre-COVID to post-COVID phases to gain insights into COVID-19 treatment strategies and assess whether pre-COVID pharmacotherapy can predict long COVID symptoms as a health status indicator. Methods: In the Precision Medicine for more Oxygen (P4O2) COVID-19 study, 95 long COVID patients were comprehensively evaluated through post-COVID outpatient clinics and study visits. This study focused on descriptive analysis of the pharmacotherapy patterns across different phases: pre-COVID-19, acute COVID, and post-COVID. Furthermore, associations between pre-COVID medication and long COVID outcomes were analyzed with regression analyses. Results: We observed peaks in the use of certain medications during the acute infection phase, including corticosteroids and antithrombotic agents, with a decrease in the use of renin–angiotensin system inhibitors. Consistently high use of alimentary tract medications was found across all phases. Pre-COVID respiratory medications were associated with fatigue symptoms, while antiinfectives and cardiovascular drugs were linked to fewer persisting long COVID symptom categories. Conclusion: Our findings provide longitudinal, descriptive pharmacotherapy insights and suggest that medication history can be a valuable health status indicator in characterizing patients for personalized disease management strategies, considering the heterogeneous nature of long COVID.

## 1. Introduction

The coronavirus disease (COVID-19), caused by severe acute respiratory syndrome coronavirus 2 (SARS-CoV-2), rapidly became a global pandemic. Many individuals recover from the acute phase, although a significant number develop persistent symptoms, known as long COVID (LC), post-acute sequelae of COVID-19, or post-COVID syndrome. While studies report varying prevalence rates, it is estimated that approximately 10–30% of COVID-19 cases experience LC [1,2]. The World Health Organization defines LC as the persistence of symptoms for more than three months [3]. These symptoms can last for months to years, impacting quality of life and creating challenges for healthcare systems [1,4,5].

LC can include various symptoms, including fatigue, respiratory, cardiovascular, neurological, and gastrointestinal complaints, complicating diagnosis and management [4,6,7]. Current treatment strategies lack effective, approved interventions. Hypotheses regarding its pathophysiology include viral persistence, immune dysregulation, microvascular injury, and autonomic dysfunction [1,5,8]. Understanding these mechanisms is crucial for developing targeted interventions [1,5].

Precision medicine, which is meant to tailor treatments based on individual characteristics such as genetics, environment, and lifestyle, is needed for characterizing LC, considering its heterogeneity [9]. While LC is associated with all ages, risk factors identified by previous studies include severe initial COVID-19, sex (with women more likely to experience persistent symptoms), smoking status, and chronic conditions like hypertension, obesity, psychiatric disorders, and immunocompromised conditions [1,8,10].

Pre-existing medication history, or pharmacotherapy, could be a potentially relevant aspect of assessing a patient’s pre-COVID-19 health status and potential susceptibility to LC development. While pharmacotherapy is a known predictor of disease outcomes in other conditions, its impact on LC is less studied [11,12,13]. Furthermore, longitudinal pharmacotherapy data may provide insights into approaches taken in COVID-19 management. This study aimed to evaluate the pharmacotherapy of LC patients from pre-COVID to post-COVID phases and to identify potential health status predictors of LC symptoms. These findings can aid in characterizing patients for the development of targeted treatment strategies, ultimately contributing to precision medicine approaches in LC.

## 2. Methods

This study is based on data collected from LC patients who participated in the Precision Medicine for more Oxygen (P4O2) COVID-19 study, a longitudinal multi-center prospective observational cohort study. This study was approved by the medical ethical board of the Amsterdam University Medical Centers, reference number NL74701.018.20. Eligible LC patients were referred to a post-COVID outpatient clinic for persisting LC symptoms in one of the participating study hospitals. Written informed consent was obtained from all participants. An extensive study protocol description and patient characteristics were published elsewhere [7].

### 2.1. Study Design and Setting

During study visits, longitudinal data were collected from participants during pre-COVID, acute infection, post-COVID, and LC phases. The P4O2 COVID-19 dataset (n = 95) includes post-COVID outpatient care reports, computed tomography (CT) scans, pulmonary function tests, laboratory measurements, physiotherapy records, questionnaires, and biological samples. Among these data, symptom data related to LC were categorized according to persistent fatigue, respiratory, neurological, gastrointestinal, and cardiovascular complaints. During P4O2 COVID-19 study visits, self-reported medication data were collected from all LC patients. The electronic health records (EHRs) were screened to verify self-reported medication data based on physician contact letters, pharmacy notes, and medication administration records. While data on dosage, timing, and route of administration were collected, this current study is based on a relatively small patient group; therefore, the medication data were analyzed qualitatively.

### 2.2. Classification of Medication Data

**Figure 1:**
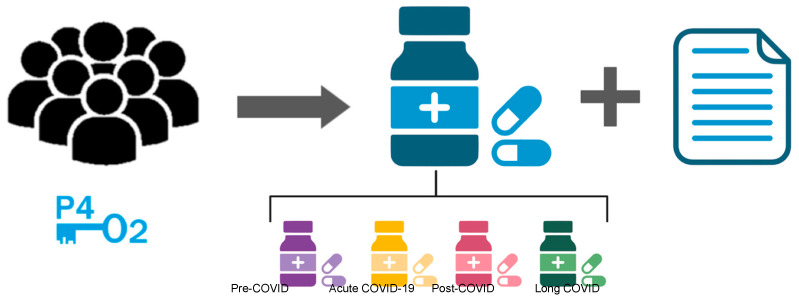
Methods visualization. Data were collected from 95 P4O2 COVID-19 study participants at 3–6 months post-infection. Pharmacotherapy was reported at four time points (pre-COVID-19, during COVID-19, post-COVID, and at 3–6 months post-infection or long COVID). Self-reported symptom data at 3–6 months were categorized into fatigue, respiratory, neurological, cardiovascular, gastrointestinal, and other complaints.

To answer the research questions, data from four time points were analyzed (see Figure 1), including the following:

- Pre-COVID;
- Acute COVID-19, during the acute infection phase;
- Post-COVID, referring to the immediate phase following recovery from acute infection;
- Long COVID, at 3–6 months post-infection.

The prescribed medications were classified according to the corresponding Anatomical Therapeutic Chemical (ATC) code. This system, which is controlled by the World Health Organization Collaborating Centre for Drug Statistics Methodology, is used to categorize the active substances into groups according to which organ or system they act upon, in addition to their chemical, pharmaceutical, and therapeutic properties. The first level of this code refers to the main anatomical group of medications and was used in regression analysis to gain insight into pharmacological categories. The second (therapeutic subgroup) was used for descriptive purposes. For research purposes, the ATC system enhances comparability among studies and allows for standardized subgroup-specific analyses of drugs that showed variation within their main anatomical group and were therefore considered relevant to LC.

The first level of ATC medication groups included the following:

- Alimentary tract (A);
- Blood and blood-forming organs (B);
- Cardiovascular system (C);
- Dermatologicals (D);
- Genito-urinary system and sex hormones (G);
- Systemic hormonal preparations, excluding sex hormones and insulins (H);
- Antiinfective for systemic use (J);
- Antineoplastic and immunomodulating agents (L);
- Musculo-skeletal system (M);
- Nervous system (N);
- Antiparasitic products, insecticides, and repellents (P);
- Respiratory system (R);
- Sensory organs (S);
- Various (V).

### 2.3. Statistical Analysis

Data analysis was performed using R software (version 4.2.1). Longitudinal data were analyzed descriptively. A logistic regression analysis was used to assess the association between the pre-COVID medication use and both the dichotomous outcome pulmonary abnormalities and the presence of the most prevalent symptom categories amongst P4O2 COVID-19 study participants, including fatigue, respiratory, and neurological complaints [7]. Results were presented as odds ratios (OR) with the corresponding 95% confidence intervals (CI) and *p*-values and considered significant if *p* < 0.05. Linear regression analyses were used to assess the association between pre-COVID pharmacotherapy and the number of persisting symptom categories. For linear regression, the results were presented as regression coefficients (β); 95% CIs and *p*-value were reported for each medication group. ATC groups were included in the regression analyses if >5 LC patients had prescribed medication in the respective class. Both logistic and linear regression models were explored with and without confounders that included age, sex, body mass index (BMI), smoking status, and acute COVID-19 severity. The World Health Organization classification for acute COVID-19 was used to categorize patients into mild, moderate, and severe disease severity groups for the acute COVID-19 severity confounding variable [7].

## 3. Results

### 3.1. Study Participant Characteristics and Pharmacotherapy Changes over Time

**Figure 2:**
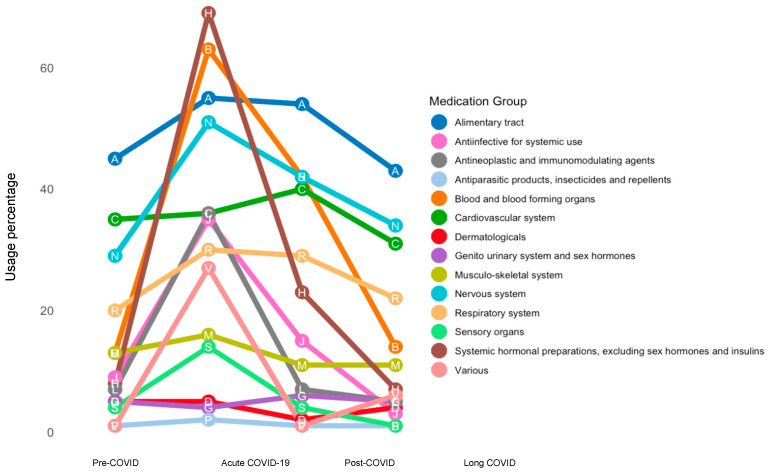
Pharmacotherapy from pre-COVID to LC. A longitudinal visualization of the percentages of pharmacotherapy use among P4O2 COVID-19 study participants, categorized according to the first-level ATC medication groups.

This study included 95 LC patients from the P4O2 COVID-19 study, with a mean age of 54.2 ± 6.2 years, and 47 (49.5%) were female. Furthermore, 35/94 (37.2%) patients were overweight and 49/94 (52.1%) were obese. Most patients were ex-smokers or never smokers, 51 (53.7%) and 40 (42.1%), respectively. Concerning the acute COVID-19 characteristics, 67 (70.6%) patients were vaccinated at least once, 61 (64.2%) had a moderate COVID-19 disease severity, 85 (89.5%) patients were hospitalized, and 27 (28.4%) were admitted to intensive care. At 3–6 months post-COVID, the most-reported persisting LC symptoms included respiratory (78.9%), neurological (68.4%), and fatigue (67.4%) complaints. The pharmacotherapy data, collected at multiple time points, revealed patterns in medication use across the pre-COVID, acute COVID-19, post-COVID, and 3–6 months post-COVID (LC) phases. Figure 2 demonstrates an overview of these longitudinal pharmacotherapy courses, and Table S1 additionally shows the exact percentages of pharmacotherapy users over time per medication group. As shown in Figure 2, the self-reported drug administration for most first-level ATC groups peaked during acute COVID-19. The largest absolute usage changes in drugs between time points according to therapeutic subgroups (ATC level 2) is shown in Table S2.

#### 3.1.1. Alimentary Tract and Metabolism

The overall use of alimentary tract and metabolism medications, which treat digestive conditions including acid reflux, irritable bowel syndrome, and peptic ulcers, remained relatively stable, while absolute changes in specific therapeutic subgroups were relatively large. Before COVID-19, 45 (47.9%) patients reported use of these medications, which increased to 55 (57.9%) during acute COVID-19, then slightly decreased to 43 (45.3%) post-COVID again. Notable increases during the acute phase were observed in subgroups A06 (drugs used for constipation), A07 (antidiarrheals and intestinal anti-inflammatory/antiinfective agents), A10 (drugs used in diabetes), and A12 (mineral supplements). Post-COVID, the usage of A07, A10, and A12 decreased, while A06 usage remained elevated, reflecting a prolonged need or lack of discontinuation.

#### 3.1.2. Blood and Blood-Forming Organs

Blood and blood-forming organs medications, including anticoagulants, saw an increase during acute COVID-19 to 63 (66.3%) from 13 (13.8%) patients pre-COVID and then decreased to 14 (14.7%) post-COVID. This change was primarily in subgroup B01 (antithrombotic agents), reflecting the treatment needs during the acute infection phase, with other changes also noted in B05 (blood substitutes and perfusion solutions).

#### 3.1.3. Cardiovascular System

Cardiovascular system medications for conditions including hypertension, heart failure, and arrhythmias remained relatively stable across time points. However, on level 2 ATC groups, increases were observed in subgroup C03 (diuretics), with an addition of 16 users during acute COVID-19. Increases were also seen in C01 (cardiac therapy) and C02 (antihypertensive drugs), while usage of C09 (agents acting on the renin–angiotensin system (RAS)) and C10 (lipid-modifying agents) declined from pre-COVID to acute COVID-19, followed by a subsequent increase to baseline use in the post-infection phase.

#### 3.1.4. Dermatologicals and Genito-Urinary System

The use of dermatologic medications, applied to conditions like eczema and psoriasis, remained stable, with usage around 5% across all time points. Similarly, medications for the genito-urinary system and sex hormones, including treatments for urinary tract infections and hormonal therapies, were consistently low, fluctuating between four (4.2%) and six (6.3%) patients.

#### 3.1.5. Systemic Hormonal Preparation and Antiinfectives for Systemic Use

Systemic hormonal preparations, excluding sex hormones and insulins, saw a notable increase during acute COVID-19, with usage rising to 69 (72.6%) patients, reflecting a significant role of corticosteroids in managing COVID-19 symptoms. Post-COVID, use dropped to seven (7.4%) patients, in which subgroup H02 (corticosteroids) showed the most distinct change between time points across all medication groups. Antiinfectives for systemic use, including antibiotics and antivirals, peaked at 35 (36.8%) patients during acute COVID-19 and then decreased to 3 (3.2%) patients post-COVID.

#### 3.1.6. Antineoplastic and Immunomodulating Agents and Musculo-Skeletal System

Antineoplastic and immunomodulating agents, used in cancer treatment and for autoimmune diseases, increased to 36 (37.9%) patients during acute COVID-19, then decreased to 5 (5.3%) post-COVID, driven primarily by subgroup L04 (immunosuppressants). Musculo-skeletal system medications remained consistent, with a slight increase to 16 (16.8%) patients during acute COVID-19 and stabilization post-COVID, with notable changes in level 2 ATC group M03 (muscle relaxants).

#### 3.1.7. Nervous System and Respiratory System

Nervous system medications, including analgesics, antiepileptics and antidepressants, increased to 51 (53.7%) patients during acute COVID-19 and remained elevated at 34 (35.8%) post-COVID. The analgesics subgroup (N02) particularly peaked during the acute phase. The use of respiratory system drugs, such as those for asthma and COPD, increased to 30 (31.6%) patients during acute COVID-19, with a slight decrease to 22 (23.2%) patients post-COVID. This was primarily due to the increased use of cough and cold medications (R05).

#### 3.1.8. Sensory Organ and Various Medications

For sensory organ medications, particularly ophthalmological drugs (S01), use peaked at 14 patients (14.7%) during acute COVID-19 and then decreased to 1 (1.1%) patient post-COVID. The various medications category, including general nutrients (V06) and contrast media (V08), peaked at 27 (28.4%) patients during acute COVID-19, followed by a decrease post-COVID.

Overall, during acute COVID-19, there was an increase in medication use across most therapeutic groups, particularly in anticoagulants, corticosteroids, and respiratory medications, reflecting the intensive management required during the infection. Post-COVID, medication usage generally decreased, with most returning close to pre-COVID levels, although some, like drugs for constipation and nervous system medications, remained slightly elevated. Findings collectively demonstrate that the acute phase saw the most significant shifts in drug administration, with stabilization occurring afterwards.

### 3.2. Regression Analyses

The regression analyses explored associations between pre-COVID medication use and LC outcomes, focusing on pulmonary abnormalities, fatigue, respiratory, and neurological symptoms, and the number of LC symptom categories. The most relevant results of the regression analyses are summarized in Table 1. Supplementary Tables S3–S7 show all identified associations. For the regression analyses, the following ATC groups were not included, because n < 5: antiparasitic products, insecticides and repellents, sensory organs, and various.

#### 3.2.1. Fatigue Symptoms

In the context of fatigue symptoms, LC patients with pre-COVID use of respiratory system medications demonstrated significantly higher odds for fatigue symptoms compared to those without pre-COVID respiratory system treatments (adjusted OR 5.74, 95% CI: 1.16–28.54, *p* = 0.03). The most prevalent pre-COVID category of respiratory medications was for obstructive airway disease (n = 15). The cardiovascular system group users presented an adjusted OR of 0.35 (95% CI: 0.12–1.06, *p* = 0.06), suggesting less long COVID fatigue symptoms in patients with such pre-COVID treatments. On the contrary, the antiinfective for systemic usage group had a lower adjusted OR of 0.22 (95% CI: 0.04–1.23, *p* = 0.09), which, while not statistically significant, potentially demonstrates a reduced occurrence of fatigue symptoms among users.

#### 3.2.2. Respiratory Symptoms

Regarding the persistence of respiratory symptoms, none of the pre-COVID medication groups showed strong associations based on the results in Table S4. The user group of respiratory system medication had higher odds for pulmonary symptoms compared to LC patients without pre-COVID respiratory medication (adjusted OR 2.67, 95% CI: 0.51–14.00, *p* = 0.25), reflecting a possible association with these symptoms.

#### 3.2.3. Neurological Symptoms

For neurological symptoms, LC patients with pre-COVID cardiovascular system treatments had lower odds compared to those without (adjusted OR 0.39, 95% CI: 0.13–1.14, *p* = 0.09), suggesting fewer neurological symptoms. LC patients with pre-COVID antiinfectives for systemic use had significantly lower odds compared with LC patients without such treatments (adjusted OR 0.11, 95% CI: 0.02–0.66, *p* = 0.02).

#### 3.2.4. The Number of Persisting Symptom Categories

The linear regression analysis of the number of LC symptom categories demonstrated some trends. The users of the pre-COVID cardiovascular system treatments had significantly fewer persistent symptom categories compared to those who did not use cardiovascular system drugs (adjusted β −0.76, 95% CI: −1.49–−0.03, *p* = 0.04).

The most commonly used therapeutic subgroups were N09 (RAS agents) and N10 (lipid-modifying therapies). The antiinfective for systemic user group also showed a significant adjusted β of −1.21 (95% CI: −2.40–−0.03, *p* = 0.046), suggesting an association with fewer symptom categories.

#### 3.2.5. Pulmonary Radiological Abnormalities

The analysis of pulmonary radiological abnormalities shows varying odds ratios across medication groups, with the adjusted ORs generally aligning closely with the unadjusted ones. While not significant, the respiratory system group users exhibited higher odds (adjusted OR 5.04, 95% CI: 0.73–34.87, *p* = 0.10) for radiological abnormalities compared with LC patients that did not have pre-COVID respiratory medication. The nervous system group showed a lower adjusted OR of 0.29 (95% CI: 0.09–0.97, *p* = 0.045), suggesting that these patients had fewer pulmonary radiological abnormalities. The most frequently prescribed subgroups were N02 (analgesic drugs) and N05 (psycholeptics drugs).

## 4. Discussion

The objectives of this study were to assess longitudinal pharmacotherapy patterns during different phases related to COVID-19 and to investigate the role of pre-COVID pharmacotherapy as a potential pre-COVID health status predictor for LC outcomes. This study showed an increase in overall medication usage during the acute COVID-19 phase, mostly in corticosteroids and antithrombotics, followed by an overall decrease to pre-COVID pharmacotherapy usage. Additionally, a decrease in the use of RAS inhibitors was observed during acute COVID-19, while the use of alimentary tract medications remained relatively high across all time points. These specific therapeutic subgroups provide insights into COVID-19 treatment strategies and suggest that pre-existing health conditions, as indicated by medication usage, are potentially linked to LC.

### 4.1. Key Pharmacotherapy Trends

The increase in blood and blood-forming organ medications, particularly anticoagulants, during the acute phase aligns with known COVID-19 complications, such as coagulopathies [1]. SARS-CoV-2 binds to angiotensin-converting enzyme 2 (ACE2) receptors, disrupting RAS balance and potentially leading to endothelial dysfunction, vascular inflammation, and heightened thrombotic risk [14,15]. This mechanism may explain the increased need for antithrombotic treatments during the acute phase, emphasizing the importance of managing thrombotic risk in COVID-19 patients, even post-recovery. It was found that anticoagulation therapy can lead to improved patient outcomes in hospitalized COVID-19 patients [16]. Moreover, anticoagulation therapy before and during the acute infection led to reduced mortality in severe COVID-19 patients [17]. The use of anticoagulants, particularly heparins, has been crucial in reducing the risk of systemic thrombosis in severe COVID-19 [18]. The identified relations between the chronic use of anticoagulants and COVID-19 outcomes are inconsistent [19]. As patients recover from COVID-19, we saw a decrease in the use of blood and blood-forming organ medications, indicating a potential reduction in thrombotic risks post-recovery. Although studies have shown that patients with COVID-19 who had superficial vein thrombosis (VTE) and stopped anticoagulation therapy after at least three months had a low incidence of recurrent VTE, it is still essential to maintain ongoing vigilance and manage thrombotic risks to prevent potential complications even after the acute phase of recovery [20].

The use of systemic hormonal preparations, mostly corticosteroids, peaked during the acute COVID-19 phase, reflecting their role in controlling severe inflammatory responses. The significant reduction in usage post-COVID indicates that these medications were predominantly used for short-term management during the height of the infection. A meta-analysis has shown that corticosteroids are effective in reducing mortality in critically ill COVID-19 patients compared with usual care or placebo [21]. Furthermore, corticosteroids are frequently prescribed as an adjuvant therapy for acute respiratory distress syndrome in general, because of their anti-inflammatory properties [22]. However, prolonged use (>10 days) of corticosteroid therapy was associated with higher mortality [23].

Cardiovascular medications, including diuretics and antihypertensives, saw a slight increase during acute COVID-19, which is consistent with managing conditions like myocarditis and thromboembolic events. The subsequent decrease in usage suggests a resolution of acute issues for many patients, though chronic conditions likely require ongoing management. A Swedish cohort study demonstrated that the initiation of all antihypertensive medicines increased during acute COVID-19. It was proposed that this increase is associated with COVID-19-related hypertension or more frequent hypertension diagnosis due to increased health care consultancy, which could contribute to the findings of our study [24]. The use of agents acting on the RAS, among others, declined during the acute infection phase. In a study that assessed whether the discontinuation of chronic RAS inhibition treatment influences COVID-19, based on the rationale that SARS-CoV-2 cell entry depends on ACE2 that can be upregulated by these drugs, it was found that discontinuation of RAS-inhibition in COVID-19 had no significant effect on the maximum severity of COVID-19. However, they mentioned that RAS inhibition may lead to a better recovery and suggested that decisions on treatment continuation or discontinuation should be made on an individual level [25]. Furthermore, a protective effect of RAS inhibition on COVID-19 hospitalization and mortality was found among patients with pharmaceutically treated hypertension [26].

Finally, our analysis revealed consistently high usage of alimentary tract medications throughout the COVID-19 timeline, suggesting a stable need for managing gastrointestinal-related conditions. This could reflect both pre-existing disorders and new issues arising during or after COVID-19. The manifestation of alimentary symptoms, such as poor appetite, diarrhea, nausea, and abdominal pain, in COVID-19 patients emphasizes the importance of understanding and managing gastrointestinal issues in the context of the disease [27]. While in the P4O2 COVID-19 cohort gastrointestinal complaints were not among the most frequently mentioned persistent symptoms [7], this can indicate that these complaints were managed or that such conditions potentially remain a concern even after acute recovery.

### 4.2. Associations with LC Symptoms

The regression analysis revealed several key associations between medication groups and LC symptoms. However, while these associations are noteworthy, this study’s observational nature limits causal inference, and potential confounding factors must be considered.

Respiratory system medications were associated with an increased risk of persistent fatigue, demonstrating the complexity of LC symptoms. This association may be influenced by underlying respiratory conditions, as indicated by pre-COVID medication use for obstructive airway disease that is particularly used in patients with chronic obstructive pulmonary disease (COPD). While the prescription of this medication category can lead to decreased fatigue in COPD patients, these patients may have a higher level of pre-existing fatigue compared to healthy individuals, possibly explaining this association [28,29].

Antiinfective medications showed significant protective effects against neurological symptoms and reduced the number of symptom categories. This could potentially result from the early management of infections, preventing extensive inflammation and immune responses contributing to LC [30]. The protective effects of antineoplastic and immunomodulating agents against neurological symptoms may suggest that immune modulation can also impact specific LC outcomes. While these associations should be interpreted with caution due to unclear underlying mechanisms, supporting evidence indicates that conditions with pre-existing inflammation, such as seasonal allergies and autoimmune diseases, are indeed linked to an increased risk of LC when adjusted for the severity of acute COVID-19 [31].

Cardiovascular medications were associated with a reduction in the number of LC symptom categories. While it was demonstrated that pre-existing cardiovascular disease can lead to poor COVID-19 outcomes, a study that focused on the effect of diuretics prior to COVID found no effect on the prognosis of COVID-19 [32,33]. A population-based case-control study in the UK showed that antihypertensive therapy including angiotensin-converting enzyme inhibitors, angiotensin receptor blockers, beta-blockers, calcium-channel blockers, thiazide diuretics, and other antihypertensive drugs is not associated with an increased risk of COVID-19 diagnosis or mortality, as most antihypertensive classes showed negative associations with COVID-19 diagnosis [34]. While acute COVID-19 severity is linked to LC development [35], studies concerning cardiovascular LC outcomes are still lacking.

Nervous system medications were associated with less pulmonary radiological abnormalities. Among the most prescribed nervous system drugs were analgesics and psycholeptics drugs including anxiolytics. The relation between anxiety and LC in general has been established. While there is no evidence of associations between pre-existing neurological drug therapies and LC outcomes, it is known that pre-existing psychiatric disorders are associated with LC development [31,36].

### 4.3. Limitations and Future Directions

This study has several strengths, including its longitudinal design, comprehensive data collection, and the use of standardized ATC codes for medication classification, which enhanced the comparability of findings among studies. However, the reliance on self-reported medication usage introduces the possibility of recall bias that is influenced by a variety of factors, although efforts were made to validate these data with EHRs [37]. In another study, it was found that self-reported medication and prescribing data agreed with each other across a wide variety of medication groups [38]. As with all LC studies, interpreting findings requires consideration of the LC patient profiles involved. Our study cohort recruited patients from post-COVID outpatient clinics and mainly included patients with a history of moderate COVID-19 and high hospitalization rates (89.5%). Since LC can also develop following mild infections, our findings require further research to confirm the potential generalizability across all LC cases. Furthermore, the observational design limits the ability to identify causality, and residual confounding cannot be ruled out despite adjustments for known confounders. Additionally, the sample size (N = 95) may limit generalizability and the power to detect smaller effects and interactions in regression analyses. This sample size was based on the availability of eligible patients referred to outpatient clinics with persistent symptoms and, despite its limitations, provided preliminary insights into pharmacotherapy patterns in LC, given this study’s longitudinal observational design.

Future research should focus on validating these findings in larger, more diverse populations globally and on exploring the biological mechanisms underlying these associations. Incorporating pharmacogenomics could provide deeper insights into how genetic profiles interact with medication use, further refining personalized treatment strategies. Additionally, it was shown that COVID-19 severity was associated with significant differential gene expression for several genes involved in drug-metabolizing enzymes and membrane transporters, including upregulation in *CYP2C9* and *CYP2C19* [39]. Longitudinal studies are also needed to understand the persistence of symptoms and the long-term effects of COVID-19, offering a pathway to improved management and care for affected individuals.

Concluding, this study demonstrated that from pre-to post-COVID, medication usage peaked during acute infection based on self-reported medication data. The most notable increases were in ATC therapeutic subgroups of corticosteroids and antithrombotics. Furthermore, a decrease in the use of RAS inhibitors was observed during acute COVID-19, and alimentary tract medication remained relatively high across all time points while most groups declined back to pre-COVID usage percentages. This study revealed associations between pre-COVID medication use and LC outcomes. While these associations need to be interpreted with caution, they suggest that medication history can potentially be a valuable health status tool to identify subsets of LC patients. By integrating precision medicine approaches, ultimately healthcare providers can develop more effective treatment plans tailored to individual patient histories, potentially improving outcomes for those suffering from LC.

## Figures and Tables

**Table 1:** Pre-COVID medication associations with persisting LC symptoms, the number of LC symptoms, and pulmonary radiological abnormalities. Logistic regression was used for associations with pulmonary abnormalities as well as fatigue, respiratory, and neurological symptom categories, while a linear regression model was used for the number of persisting LC symptom categories.

Fatigue Symptoms						
		Unadjusted			Adjusted *	
Medication Group	OR	95% CI	*p*-value	OR	95% CI	*p*-value
Cardiovascular system	0.56	0.23–1.36	0.20	0.35	0.12–1.06	0.06
Antiinfective for systemic use	0.20	0.05–0.85	0.03	0.22	0.04–1.23	0.09
Respiratory system	5.48	1.18–25.41	0.03	5.74	1.16–28.54	0.03
**Respiratory symptoms**						
		**Unadjusted**			Adjusted *	
**Medication Group**	**OR**	**95% CI**	***p*** **-value**	**OR**	**95% CI**	***p*** **-value**
Respiratory system	2.89	0.61–13.69	0.18	2.67	0.51–14.00	0.25
**Neurological symptoms**						
		**Unadjusted**			Adjusted *	
**Medication Group**	**OR**	**95% CI**	***p*** **-value**	**OR**	**95% CI**	***p*** **-value**
Cardiovascular system	0.51	0.21–1.25	0.14	0.39	0.13–1.14	0.09
Antiinfective for systemic use	0.10	0.02–0.52	0.01	0.11	0.02–0.66	0.02
**Number of symptom categories**						
		**Unadjusted**			Adjusted *	
**Medication Group**	**β**	**95% CI**	***p*** **-value**	**β**	**95% CI**	***p*** **-value**
Cardiovascular system	−0.50	−1.18–0.19	0.15	−0.76	−1.49–−0.03	0.04
Antiinfective for systemic use	−1.59	−2.68–−0.50	0.01	−1.21	−2.40–−0.03	0.046
**Pulmonary radiological abnormalities**						
		**Unadjusted**			Adjusted *	
**Medication Group**	**OR**	**95% CI**	***p*** **-value**	**OR**	**95% CI**	***p*** **-value**
Nervous system	0.50	0.18–1.39	0.18	0.29	0.09–0.97	0.045
Respiratory system	2.79	0.58–13.39	0.20	5.04	0.73–34.87	0.10

* Adjusted for confounders including age, sex, BMI, smoking status, and acute COVID-19 severity (WHO score).

## Data Availability

The data presented in this study are available on request from the corresponding authors.

## Supplementary Materials

The following supporting information can be downloaded at: https://www.mdpi.com/article/10.3390/biomedicines12122694/s1, Table S1: Descriptive longitudinal pharmacotherapy data; Table S2: Top 50 absolute changes in pharmacotherapy according to ATC level 2 groups between different time points; Table S3: Pre-COVID medication associations with fatigue symptoms; Table S4: Pre-COVID medication associations with pulmonary symptoms; Table S5: Pre-COVID medication associations with neurological symptoms; Table S6: Pre-COVID medication associations with the number of symptoms categories; Table S7: Pre-COVID medication associations with pulmonary radiological abnormalities.
