# Supplementary material for: Pharmacotherapy from Pre-COVID to Post-COVID: Longitudinal Trends and Predictive Indicators for Long COVID Symptoms
Source: Biomedicines. 2024 Nov 26;12(12):2694. doi: 10.3390/biomedicines12122694 (PMC11673229; doi:10.3390/biomedicines12122694)
Supplement: Supplementary file 1 [file biomedicines-12-02694-s001.zip › biomedicines-3291398-supplementary.pdf]

Appendices

Table S1. Descriptive longitudinal pharmacotherapy data

Medication group	Pre-COVID	Acute COVID-19	Hospital discharge	3-6 months post-COVID
Alimentary tract	45 (47.9%)	55 (57.9%)	54 (56.8%)	43 (45.3%)
Blood and blood forming organs	13 (13.8%)	63 (66.3%)	42 (44.2%)	14 (14.7%)
Cardiovascular system	35 (37.2%)	36 (37.9%)	40 (42.1%)	31 (32.6%)
Dermatologicals	5 (5.3%)	5 (5.3%)	2 (2.1%)	4 (4.2%)
Genito urinary system and sex hormones	5 (5.3%)	4 (4.2%)	6 (6.3%)	5 (5.3%)
Systemic hormonal preparations, excluding sex hormones and insulins	8 (8.5%)	69 (72.6%)	23 (24.2%)	7 (7.4%)
Antiinfective for systemic use	9 (9.6%)	35 (36.8%)	15 (15.8%)	3 (3.2%)
Antineoplastic and immunomodulating agents	7 (7.4%)	36 (37.9%)	7 (7.4%)	5 (5.3%)
Musculo-skeletal system	13 (13.8%)	16 (16.8%)	11 (11.6%)	11 (11.6%)
Nervous system	29 (30.9%)	51 (53.7%)	42 (44.2%)	34 (35.8%)
Antiparasitic products, insecticides and repellents	1 (1.1%)	2 (2.1%)	1 (1.1%)	1 (1.1%)
Respiratory system	20 (21.3%)	30 (31.6%)	29 (30.5%)	22 (23.2%)
Sensory organs	4 (4.3%)	14 (14.7%)	4 (4.2%)	1 (1.1%)
Various	1 (1.1%)	27 (28.4%)	1 (1.1%)	6 (6.3%)

Table S2. Top 50 absolute changes in pharmacotherapy according to ATC level 2 groups between different time points.

ATC level 2 group	Time point 1	Time point 2	Change in the number of users
H02	Before infection	During infection	67
H02	During infection	Study visit 1	-67
B01	Before infection	During infection	52
B01	During infection	Study visit 1	-51
N02	Before infection	During infection	34
B05	Before infection	During infection	31
L04	During infection	Study visit 1	-31
B01	After infection	Before infection	-30
B05	During infection	Study visit 1	-30
N02	During infection	Study visit 1	-30
L04	Before infection	During infection	29
J01	During infection	Study visit 1	-28
A06	Before infection	During infection	27
A10	Before infection	During infection	27
A10	During infection	Study visit 1	-26
A06	During infection	Study visit 1	-24
J01	Before infection	During infection	23
A12	Before infection	During infection	21
V08	Before infection	During infection	21
A07	Before infection	During infection	20
R05	Before infection	During infection	20
R05	During infection	Study visit 1	-20
A07	During infection	Study visit 1	-19
V08	During infection	Study visit 1	-19
A12	During infection	Study visit 1	-18
N01	During infection	Study visit 1	-18
C03	During infection	Study visit 1	-17
H02	After infection	Before infection	-17
N01	Before infection	During infection	17
V06	Before infection	During infection	17
C03	Before infection	During infection	16
N05	During infection	Study visit 1	-16
C01	During infection	Study visit 1	-15
N05	Before infection	During infection	15
V06	During infection	Study visit 1	-15
C01	Before infection	During infection	13
S01	During infection	Study visit 1	-13
C02	During infection	Study visit 1	-11
S01	Before infection	During infection	11
C02	Before infection	During infection	9
M03	Before infection	During infection	9
M03	During infection	Study visit 1	-9
N02	After infection	Before infection	-9

A06	After infection	Before infection	-8
C09	Before infection	During infection	-8
C10	Before infection	During infection	-8
N06	During infection	Study visit 1	8
A02	After infection	Before infection	-7
J05	During infection	Study visit 1	-7
R05	After infection	Before infection	-7

Table S3. Pre-COVID medication associations with fatigue symptoms.

Fatigue symptoms						
	**Unadjusted**			**Adjusted***		
**Medication Group**	**OR**	**95% CI**	**p-value**	**OR**	**95% CI**	**p-value**
Alimentary tract	1.31	0.55 - 3.13	0.55	1.39	0.51 - 3.74	0.52
Blood and blood forming organs	0.49	0.15 - 1.62	0.24	0.37	0.10 - 1.38	0.14
Cardiovascular system	0.56	0.23 - 1.36	0.20	0.35	0.12 - 1.06	0.06
Dermatologicals	1.93	0.21 - 18.08	0.56	1.31	0.12 - 13.93	0.82
Genito urinary system and sex hormones	1.93	0.21 - 18.08	0.56	1.49	0.13 - 16.74	0.75
Systemic hormonal preparations, excluding sex hormones and insulins	1.45	0.28 - 7.64	0.66	0.95	0.16 - 5.85	0.96
Antiinfective for systemic use	0.20	0.05 - 0.85	0.03	0.22	0.04 - 1.23	0.09
Antineoplastic and immunomodulating agents	1.19	0.22 - 6.50	0.84	1.17	0.19 - 7.37	0.87
Musculo-skeletal system	1.06	0.30 - 3.78	0.92	1.29	0.33 - 5.12	0.71
Nervous system	1.72	0.64 - 4.64	0.28	1.47	0.48 - 4.50	0.50
Respiratory system	5.48	1.18 - 25.41	0.03	5.74	1.16 - 28.54	0.03

Adjusted for confounders including age, sex, BMI, smoking status, acute COVID-19 severity (WHO score).

Table S4. Pre-COVID medication associations with pulmonary symptoms.

Pulmonary symptoms						
	Unadjusted			Adjusted*		
**Medication Group**	**OR**	**95% CI**	**p-value**	**OR**	**95% CI**	**p-value**
Alimentary tract	1.16	0.43 - 3.12	0.77	1.27	0.40 - 4.07	0.69
Blood and blood forming organs	1.57	0.32 - 7.75	0.58	0.89	0.15 - 5.33	0.90
Cardiovascular system	0.86	0.31 - 2.37	0.77	0.59	0.17 - 2.01	0.40
Dermatologicals	0.38	0.06 - 2.45	0.31	0.17	0.02 - 1.63	0.13
Genito urinary system and sex hormones	1.09	0.12 - 10.29	0.94	0.45	0.04 - 5.55	0.53
Systemic hormonal preparations, excluding sex hormones and insulins	1.99	0.23 - 17.15	0.53	1.49	0.15 - 15.14	0.74
Antiinfective for systemic use	0.29	0.07 - 1.20	0.09	0.75	0.13 - 4.43	0.75
Antineoplastic and immunomodulating agents	1.68	0.19 - 14.79	0.64	1.10	0.11 - 11.43	0.94
Musculo-skeletal system	0.55	0.15 - 2.03	0.37	0.43	0.09 - 2.02	0.28
Nervous system	1.44	0.47 - 4.43	0.53	1.27	0.34 - 4.80	0.73
Respiratory system	2.89	0.61 - 13.69	0.18	2.67	0.51 - 14.00	0.25

*Adjusted for confounders including age, sex, BMI, smoking status, acute COVID-19 severity (WHO score).

Table S5. Pre-COVID medication associations with neurological symptoms.

Neurological symptoms						
	**Unadjusted**			**Adjusted***		
**Medication Group**	**OR**	**95% CI**	**p-value**	**OR**	**95% CI**	**p-value**
Alimentary tract	0.98	0.41 - 2.35	0.96	1.08	0.41 - 2.87	0.88
Blood and blood forming organs	1.00	0.28 - 3.57	1.00	0.81	0.21 - 3.11	0.76
Cardiovascular system	0.51	0.21 - 1.25	0.14	0.39	0.13 - 1.14	0.09
Dermatologicals	1.84	0.20 - 17.19	0.59	1.75	0.18 - 17.47	0.64
Genito urinary system and sex hormones	0.65	0.10 - 4.14	0.65	0.41	0.05 - 3.13	0.39
Systemic hormonal preparations, excluding sex hormones and insulins	0.72	0.16 - 3.25	0.67	0.46	0.09 - 2.34	0.35
Antiinfective for systemic use	0.10	0.02 - 0.52	0.01	0.11	0.02 - 0.66	0.02
Antineoplastic and immunomodulating agents	0.30	0.06 - 1.45	0.14	0.15	0.03 - 0.90	0.04
Musculo-skeletal system	0.67	0.20 - 2.27	0.52	0.58	0.16 - 2.14	0.41
Nervous system	0.99	0.38 - 2.55	0.98	0.76	0.26 - 2.17	0.60
Respiratory system	1.05	0.36 - 3.09	0.93	0.99	0.31 - 3.18	0.99

*Adjusted for confounders including age, sex, BMI, smoking status, acute COVID-19 severity (WHO score).

Table S6. Pre-COVID medication associations with the number of symptoms categories.

Number of symptom categories						
	Unadjusted			Adjusted*		
**Medication Group**	**β**	**95% CI**	**p-value**	**β**	**95% CI**	**p-value**
Alimentary tract	0.10	-0.57 - 0.77	0.78	0.32	-0.38 - 1.01	0.36
Blood and blood forming organs	0.05	-0.92 - 1.02	0.92	-0.24	-1.19 - 0.72	0.62
Cardiovascular system	-0.50	-1.18 - 0.19	0.15	-0.76	-1.49 - -0.03	0.04
Dermatologicals	-0.09	-1.58 - 1.40	0.91	-0.50	-1.96 - 0.96	0.50
Genito urinary system and sex hormones	-0.09	-1.58 - 1.40	0.91	-0.34	-1.95 - 1.26	0.67
Systemic hormonal preparations, excluding sex hormones and insulins	0.40	-0.80 - 1.60	0.51	0.15	-1.05 - 1.35	0.80
Antiinfective for systemic use	-1.59	-2.68 - -0.50	0.01	-1.21	-2.40 - -0.03	0.05
Antineoplastic and immunomodulating agents	0.28	-0.99 - 1.56	0.66	0.04	-1.24 - 1.31	0.95
Musculo-skeletal system	0.14	-0.83 - 1.10	0.78	0.16	-0.80 - 1.12	0.74
Nervous system	0.32	-0.40 - 1.04	0.38	0.23	-0.53 - 0.98	0.55
Respiratory system	0.34	-0.48 - 1.15	0.41	0.24	-0.58 - 1.06	0.56

*Adjusted for confounders including age, sex, BMI, smoking status, acute COVID-19 severity (WHO score).

Table S7. Pre-COVID medication associations with pulmonary radiological abnormalities.

Pulmonary radiological abnormalities						
	Unadjusted			Adjusted*		
**Medication Group**	**OR**	**95% CI**	**p-value**	**OR**	**95% CI**	**p-value**
Alimentary tract	1.18	0.44 - 3.18	0.74	0.72	0.23 - 2.27	0.58
Blood and blood forming organs	0.71	0.17 - 3.04	0.65	0.44	0.08 - 2.32	0.33
Cardiovascular system	1.43	0.49 - 4.17	0.51	1.06	0.29 - 3.89	0.93
Dermatologicals	0.63	0.05 - 7.26	0.71	1.15	0.06 - 22.85	0.93
Genito urinary system and sex hormones	0.95	0.09 - 9.68	0.97	0.98	0.05 - 21.36	0.99
Systemic hormonal preparations, excluding sex hormones and insulins	0.95	0.18 - 5.11	0.95	0.54	0.08 - 3.48	0.51
Antiinfective for systemic use	0.39	0.08 - 1.89	0.24	0.58	0.08 - 4.49	0.60
Antineoplastic and immunomodulating agents	2.00	0.23 - 17.63	0.53	1.09	0.11 - 11.00	0.95
Musculo-skeletal system	1.07	0.27 - 4.32	0.92	0.96	0.20 - 4.62	0.96
Nervous system	0.50	0.18 - 1.39	0.18	0.29	0.09 - 0.97	0.05
Respiratory system	2.79	0.58 - 13.39	0.20	5.04	0.73 - 34.87	0.10

*Adjusted for confounders including age, sex, BMI, smoking status, acute COVID-19 severity (WHO score).
